# 

**Published:** 1891-07

**Authors:** 


					﻿Entered at the Post Office at Austin, Texas, as Second Class Mall Matter.
Vol. VII.
JULY, 1891.
No. 1.
DANIEL'S
MEDICAL
JOURNAL
Subscription, $2.00 a Year, in Advance – Single Copies, Twenty-five Cents.
F. E. DANNIEL, M. D., Editor and Publisher, AUSTIN, TEXAS.
Eugene Von Boeckmann, Steam Book and Job Printer, Austin, Texas.
				

